# Health literacy and digital health information-seeking behavior – a cross-sectional study among highly educated Swedes

**DOI:** 10.1186/s12889-022-14751-z

**Published:** 2022-12-05

**Authors:** Erica Sundell, Josefin Wångdahl, Åsa Grauman

**Affiliations:** 1 Centre for Research Ethics & Bioethics, Uppsala University, Box 564, 751 22 Uppsala, Sweden; 2 Aging Research Center, Karolinska Institute and Stockholm University, Solna, Sweden; 3 Department of Public Health and Caring Sciences, Uppsala University, Uppsala, Sweden

**Keywords:** Public Health, Health literacy, C & C HL scale, Information-seeking behavior, Electronic health records, Internet use, Self-management, Sweden

## Abstract

**Background:**

The benefits of digital development in health care may be obscured by unequal opportunities to make use of digital resources. The aim of this study was to investigate the association of health literacy with I) accessing health check test results in the Patient Electronic Health Record (PAEHR), II) searching for information to better understand individual test results, and III) using the national health information online portal provided by the Swedish national health care system.

**Methods:**

This cross-sectional study included data from 434 individuals, 50–64 years old, randomly selected from the Swedish population during the year 2017 to a cohort study including health examination and a web-based survey. Health literacy was assessed at baseline using the Swedish Communicative and Critical Health Literacy scale. Digital information outcomes were assessed after three months. Adjusted odds ratios (ORs) and 95% confidence intervals (CI) for the separate outcomes were computed using logistic regression. Covariates included sex, age, education, country of birth, cardiovascular risk factors at baseline, general health, risk perception, referral, and new cardiovascular risk factors detected at health examination.

**Results:**

About a third of the participants (35%) had limited health literacy, while 65% had sufficient health literacy. Sufficient health literacy was associated with accessing the PAEHR (adjusted OR 1.81 95% CI 1.07–3.06) and use of the online national health information portal provided by the Swedish national health care system (adjusted OR 2.91 95% CI 1.13–7.52) but not with searching information to better understand individual test results (adjusted OR 1.29 0.75–2.20).

**Conclusions:**

Individuals with limited health literacy do not access their personal health information nor search for health information on the online national health information portal provided by the Swedish national health care system to the same extent as individuals with sufficient health literacy. More research is needed about how the level of health literacy relates to differences in online health information-seeking behavior and how digital health information sources and e-health services can be designed to ensure that the entire population has equal access to trustworthy and quality-ensured health information.

**Supplementary Information:**

The online version contains supplementary material available at 10.1186/s12889-022-14751-z.

## Background

The use of digital tools and media to spread health messages, offer health information and navigate among health care services is increasing globally [1]. Digital tools, such as the Patient Accessible Electronic Health Record (PAEHR) for instance, offer great promise for improving patient care and can facilitate self-management of chronic diseases [2]. Individuals use the PAEHR to acquire an overview of one’s health status, access information about health examination results [3], renew medication prescriptions, and to communicate with their health care provider [4]. However, using PAEHRs places great demands on health literacy for both patients and caregivers [5].

The World Health Organization define health literacy as “the cognitive and social skills which determine the motivation and ability of individuals to gain access to, understand and use information in ways which promote and maintain good health” [6], although numerous definitions exist [7]. Health literacy can be divided into functional, communicative- (i.e. interactive), and critical health literacy [7]. Nutbeam and Lloyd describe functional health literacy as the basic-level skills needed to obtain relevant health information and use that knowledge for specific health-related activities [7]. Communicative health literacy refers to more advanced skills needed to extract and derive meaning from health information, apply new and discriminate between different sources of information, engage in interactions with others to extend the information available, and make decisions, often in changing circumstances [7]. Critical health literacy refers to the most advanced skills needed to critically analyze information from different sources. The communicative and critical health literacy skills are related to being active in promoting one’s health, autonomy and decision making [7]. Furthermore, there is the concept of digital health literacy. It relates to a component of health literacy that involves specific skills to appraise health information from electronic sources and to use digital tools and health services [2].

People living in poor socioeconomic conditions are more likely to have limited health literacy [7–10]. Older adults and people with a different ethnicity than the majority population [8, 11] also have more limited health literacy compared to others. From a health perspective, limited health literacy is a serious concern as it is associated with, e.g., poorer self-perceived general health [9, 10, 12], impaired mental health [12], both higher use of healthcare services [13, 14], and refraining from seeking health care [12], worse medication adherence [7], lower participation in mammography screening, and poorer overall health status, as well as higher mortality rates among elderly persons [15].

Health checks are a preventive strategy to reduce cardiovascular mortality rates [16], and are especially important for individuals with low health literacy who are at a higher risk of disease [15]. However, low health literacy has predicted lower participation in health checks [17, 18]. In a previous study where health check test results were partly presented in the PAEHR, Swedish lay people experienced difficulties understanding the test results and needed further information to understand the implications and the possibilities for acting on their test results [19]. Low health literacy is associated with poorer health-related knowledge and ability to interpret health messages [15], so individuals with low health literate may face an even greater challenge to understand their test results. Furthermore, patients with limited functional health literacy have been found to be less likely to use the PAEHR [20], which may constitute a further obstacle for those with low health literately to utilizing their health check test results. To make sense, of test results, people may search for information from various sources including the internet. However, people might find unreliable or misleading information on the internet, leaving them confused or worried [21]. When it comes to health information-seeking behavior, individuals with higher health literacy seem to use more sources [22, 23], seek more health information online [24], and be more likely to get the health information they need [22]. Individuals with low health literacy may have a high capacity to find information while lacking the ability to judge the reliability of the information. Thus they may make decisions about their health that are based on incorrect information, with negative consequences for their health [25]. Health check participants have requested trustworthy educational resources online [26]. In Sweden and several other countries, the national health care system provides an online portal with quality-ensured health information to the public (e.g., Medlineplus.gov, NHS.uk) [4]. Using such resources as a source of health information is a way for the individual to ensure that the information is trustworthy and that it can be used to make informed health decisions.

To prevent the rise of new social inequality related to increased online health information and digitalization in society [27, 28], it is crucial to analyze how individuals with different health literacy level access, seek, and use health information sources. Previous studies within this field in Sweden have focused on migrants [12, 29]; other studies have been conducted in other parts of the world, or/and with patient populations. To broaden the knowledge of how health literacy is related to digital health information-seeking behavior, the present study involves a middle-aged general population. These individuals might not have had as much contact with the health care system compared with patient populations, but they are an important target group for primary prevention interventions, such as health checks. The study also applies a context-specific situation of receiving test results from a health examination, and how health information-seeking behavior and the use of PAEHR is related to that specific situation. This could add new perspectives on how health literacy matters in a health check context with a “healthy population.”

This study aimed to investigate the association of health literacy with I) accessing health check test results in the Patient Electronic Health Record (PAEHR), II) searching for information to better understand individual test results, and III) using the national health information online portal provided by the Swedish national health care system.

The study hypothesizes that health literacy matters when making sense of test results from a health check, including among highly educated Swedes, and that having sufficient health literacy increases the likelihood of using the PAEHR, searching for information and using the national health information online portal provided by the Swedish national health care system as a source for information.

## Methods

This was a cross-sectional study.

### Setting and participants

This was a secondary analysis of cross-sectional data collected through an add-on study to the Swedish CArdioPulmonary BioImage Study (SCAPIS) [30], with the overall objective of creating a cohort for the study of chronic obstructive pulmonary disease (COPD) and cardiovascular disease (CVD). Data was collected from 30,000 individuals through extensive health examinations at six study sites in Sweden. The study site in Uppsala collected data from 5000 individuals. The participants in SCAPIS were 50–64 years old, randomly selected from the Swedish population through the Swedish population register, and invited by letter and telephone calls (47% accepted the invitation to participate) [31]. Besides age, an inclusion criterion was the ability to understand spoken and written Swedish. The participants received some of the test results from the health checks as a written report including cardiovascular (CV) risk factors. Some additional test results could be found in their PAEHR [4]. In the case of clinical findings, the participants were referred to either primary or specialized health care, where they received routine care.

**Fig. 1:**
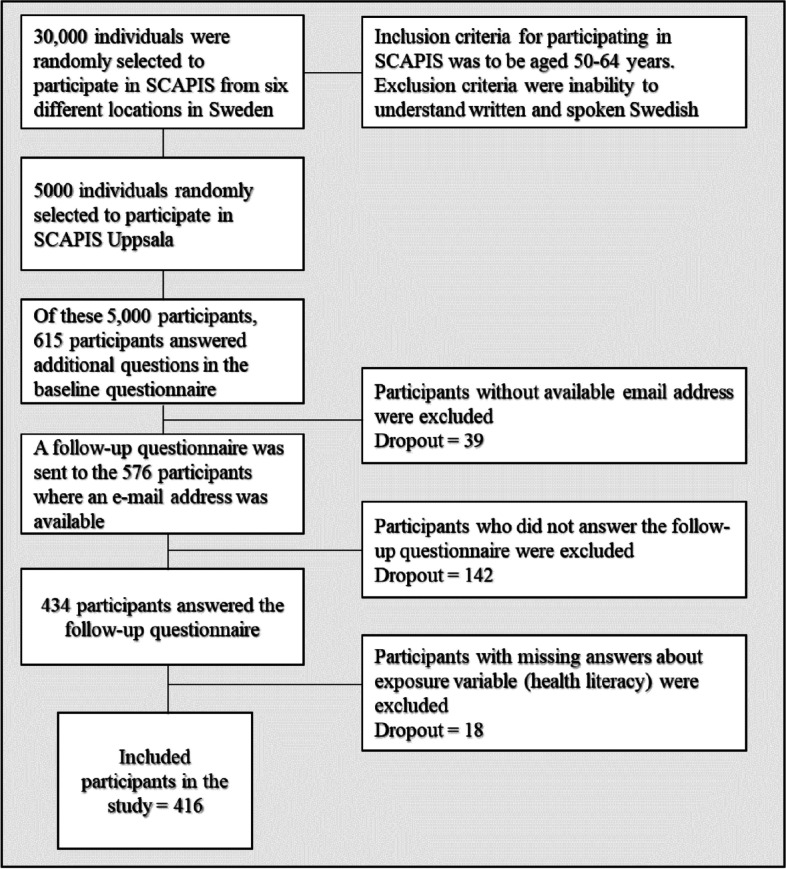
Flowchart of the study population

This add-on study to SCAPIS was enabled by adding questions to the original SCAPIS web-based questionnaire, e.g., about risk perception, mental distress, and health literacy. Studies about risk perception and mental distress have been published previously [32, 33]. Based on power calculations for the earlier studies, 615 participants was deemed a sufficient number. After 615 participants had responded to the extended questionnaire, the add-on questions were removed to not burden the participants. Three months after the 615 participants’ first visit to the test center, an online follow-up survey was e-mailed to everyone who provided their e-mail address (*n* = 576), including questions about clinical findings and information-seeking behavior. Two reminders were sent out at approximately two-week intervals. In total, 434 participants answered the follow-up questionnaire (response rate = 70%). A flowchart of the study population is shown in Fig. 1. The data collection with these additional questions were conducted February–March 2017.

Sweden is a country with public funded health care. The Swedish national health care system provides an online national portal for health information and e-services, called Healthcare Guide 1177 (available at: https://www.1177.se). It is a quality-ensured website gathering comprehensive information for the public about symptoms, diseases, treatments, rules, and patients’ rights. The information is available in multiple languages. Furthermore, the portal provides information about self-management of health and illness. After authentication, the Healthcare Guide 1177 also gives individuals access to personalized e-health services where they can make contact with health care providers and view their PAEHR [4]. The county where this study was conducted was the first county to implement PAEHRs in Sweden, back in 2012 [4].

### Measures

#### Outcome variables

Accessing the PAEHR was assessed using the question: Since your participation in SCAPIS, have you logged in to the PAEHR to view your test results? Yes/no.

Information-seeking was assessed using the question: Have you since your participation in SCAPIS searched for information to better understand the meaning of your test results? Yes/no.

If the participants answered yes to the previous question, they were asked where they searched for information. Multiple responses were possible. The alternatives were primary health care center / specialist physician/ Google / relatives, friends or acquaintances/newspapers or books / other. They could also choose the Healthcare Guide 1177 [34]. Source of information was dichotomized (Healthcare Guide 1177 = 1, all other alternatives = 0).

#### Exposure variable

Health literacy (HL) was measured using the validated Swedish version of The Communicative and Critical Health Literacy scale (S – C & C HL scale) consisting of five items [35]. The Swedish population have a high literacy level due to nine years’ mandatory schooling, so functional health literacy appears to be less of an issue. The Swedish version is a translated version of the C & C HL scale, originally developed in Japan [36]. The S – C & C HL scale is a valid and reliable instrument to use in the Swedish population [35, 37], Cronbach α = 0.87. Three items focus on an individual’s ability to retrieve information from a variety of sources, select relevant information and understand and share information with others (i.e. communicative health literacy). Two items focus on an individual’s ability to assess the credibility of information and, with the help of information, to be able to plan and decide what they need to do in order to improve their health (i.e., critical health literacy). All items are answered on a five-point ordinal scale ranging from strongly agree (= 1) to strongly disagree (= 5). A higher number indicates higher health literacy. According to the manual of the instrument [38], participants who answered “strongly agree” or “agree” on all items were categorized as having sufficient HL (n = 269); those who answered “partially agree” on all or some of the items (while answering “strongly agree”, or “agree” on the rest) were categorized as having problematic HL (*n* = 132) and those who answered “strongly disagree” or “disagree” on any item were categorized as having inadequate HL (*n* = 15). Since only 15 individuals were categorized as having inadequate health literacy, this category was merged with problematic health literacy and defined as limited health literacy in the text. No overall health literacy index score was calculated.

#### Covariates

Sociodemographic variables included sex, age, education level (graduated from primary school, high school or university), and whether the individual was born in Sweden. Medical background was assessed by asking about treatment for or diagnosis of cardiovascular disease (CVD), diabetes, hypertension, or high cholesterol before participating in SCAPIS. Questions about referral to a primary health care center (PHCC) or a hospital due to findings in SCAPIS were included, as were questions related to diagnosis of hypertension, high cholesterol, or coronary artery stenosis. Self-perceived cardiovascular risk was assessed with the question: “Compared to similar others of the same age and sex as you, how do you perceive your risk of experiencing a myocardial infarction within the next ten years? The answer was collapsed into two categories: lower or same as others (1–4 points), and higher than others (5–7 points). General health was collapsed into excellent/very good and good/somewhat good/poor.

### Statistical analyses

Participants with no missing data on the exposure variable were included in the analysis (*n* = 416). Since this is a secondary analysis of previously collected data, no power calculation for this study aim was conducted before the analysis. However, according to a rule of thumb for regression analyses, 15 individuals are needed for each degree of freedom [39]. The analyses of this study included a maximum 15 degrees of freedom; hence a minimum of 225 individuals were deemed sufficient for two of the outcomes. However, for the third, seeking information on the Healthcare Guide 1177, only a subsample (*n* = 117) that responded that they searched for information was included. The analysis could therefore not adjust for all covariates at once. Descriptive statistics are presented with mean and standard deviation for continuous variables and as frequencies for categorical variables. The association of health literacy with I) Accessing the PAEHR, II) Searching for information to better understand individual test results, and III) Seeking information on the national health information online portal system Health care guide 1177 was estimated as odds ratios (ORs) and 95% confidence intervals (CI) using logistic regression analyses. Confounders were explored and selected based on directed acyclic graphs (DAGs). Model 1 included age, sex, education, and country of birth. Model 2 additionally included general health, cardiovascular disease, diabetes, hypertension, and high cholesterol. Model 3 included model 1, 2 and cardiovascular risk perception. For associations II and III the model also included being referred to health care due to findings in SCAPIS, being diagnosed with hypertension or high cholesterol, and being diagnosed with coronary artery stenosis. All reported *p* values were two-tailed and statistical significance was defined as *p* < 0.05. All analyses were performed using JASP version 0.16.1.

## Results

Descriptive statistics of the participants in the follow-up sample with data on the exposure variable (*n* = 416) are presented in Table 1, in total and stratified by exposure. Half of the participants had a university education and 90% were born in Sweden. Of the participants, 35% had limited, i.e., problematic (31.7%) or inadequate (3.6%) health literacy, while 65% had sufficient health literacy. A larger proportion of participants with sufficient health literacy perceived their general health as very good or excellent, compared with those who had limited health literacy.

**Table 1:** Descriptive data of participants (416). Continuous variables are expressed as mean (standard deviation), categorical as number (percentages), *n* = 416

		Health literacy n (%)	
	Total n (%)	Limited^a^	Sufficient
Total	416 (100)	147 (35.3)	269 (64.7)
Age in years, mean (SD)	57.9 (4.4)	58.6 (4.4)	57.6 (4.3)
Female	222 (53.4)	75 (51.0)	147 (54.6)
Born in Sweden	374 (89.9)	127 (86.4)	247 (91.8)
Highest educational level: university	211 (50.7)	55 (37.4)	156 (58.0)
Self-reported CVD	26 (6.3)	13 (8.8)	13 (4.9)
Self-reported hypertension	89 (21.4)	36 (24.5)	53 (19.8)
Self-reported high blood pressure	47 (11.3)	12 (8.2)	35 (13.1)
Self-reported diabetes	15 (3.6)	8 (5.4)	7 (2.6)
Very good or excellent general health	205 (49.3)	56 (38.1)	149 (55.4)
Perceived cardiovascular risk higher than others	92 (22.7)	38 (27.0)	54 (20.4)
Referred to primary health care center	83 (20.0)	29 (19.7)	54 (20.1)
Referred to hospital	60 (14.4)	23 (15.6)	37 (13.8)
Diagnosed with hypertension	18 (4.3)	12 (8.2)	6 (2.2)
Diagnosed with high cholesterol	9 (2.2)	5 (3.4)	4 (1.5)
Diagnosed with coronary artery stenosis	15 (3.6)	6 (4.1)	9 (3.3)
Accessed test results in PAEHR	319 (78.0)	98 (69.0)	221 (82.8)
Searched for information to better understand test results	117 (29.0)	37 (26.6)	80 (30.2)
If yes, searched for information to better understand test results, what source used:			
Health Care Guide177	49 (41.9)	11 (29.7)	38 (47.5)
Google	74 (63.2)	22 (59.5)	52 (65.0)
Primary health care center	24 (20.5)	9 (24.3)	15 (18.8)
Physician (specialist)	6 (5.1)	3 (8.1)	3 (3.8)
Family/friends	26 (22.2)	7 (18.9)	19 (23.8)
News papers	4 (3.4)	0	4 (5.0)
Other	6 (5.1)	4 (10.8)	2 (2.5)

^a^limited = problematic and inadequate health literacy

The Cronbach α for the S – C & C HL scale in this study was 0.84. Removing one of the items reduced the Cronbach α. Of the participants, 78% had accessed their test results in the PAEHR; 83% within this group had sufficient health literacy, compared with 69% with limited health literacy. Participants with sufficient health literacy were more likely (adjusted OR 1.81), to access their PAEHR compared to participants with limited health literacy (Table 2).

**Table 2:** The association of health literacy and accessing test results in the patient electronic health record (PAEHR), OR (95% CI), *n* = 416

	Crude	Model 1	Model 2	Model 3
	OR (95% CI)	Adj. OR (95% CI)	Adj. OR (95% CI)	Adj. OR (95% CI)
Sufficient health literacy	2.16 (1.34–3.48)	1.88 (1.15–3.07)	1.81 (1.08–3.01)	1.81 (1.07–3.06)
Age (years)		0.98 (0.92–1.03)	0.98 (0.92–1.04)	0.98 (0.92–1.04)
Female		1.08 (0.67–1.75)	1.07 (0.66–1.75)	1.05 (0.64–1.73)
Born in Sweden		1.40 (0.68–2.92)	1.34 (0.63–2.84)	1.18 (0.53–2.65)
Highest educational level: university		1.67 (1.02–2.73)	1.67 (1.00–2.80)	1.61 (0.95–2.72)
Self-reported cardiovascular disease			0.27 (0.11–0.66)	0.29 (0.12–0.73)
Self-reported hypertension			0.94 (0.51–1.72)	0.98 (0.53–1.83)
Self-reported high cholesterol			1.29 (0.56–2.99)	1.24 (0.53–2.89)
Self-reported diabetes			0.58 (0.17–1.98)	0.59 (0.17–2.05)
Poor, fairly good, good general health			1.05 (0.62–1.76)	1.11 (0.64–1.92)
Perceived cardiovascular risk higher than others				0.88 (0.46–1.66)

*Adj. OR* Adjusted Odds Ratio, *CI* Confidence Interval

Twenty-nine percent of the participants reported that they had sought information to better understand their test results. However, no association with health literacy could be seen (Table 3).

**Table 3:** The association of health literacy and searching for information to better understand their individual test result, OR (95% CI), *n* = 416

	Crude	Model 1	Model 2	Model 3
	OR (95% CI)	Adj. OR (95% CI)	Adj. OR (95% CI)	Adj. OR (95% CI)
Sufficient health literacy	1.19 (0.75–1.89)	1.21 (0.75–1.94)	1.17 (0.72–1.90)	1.29 (0.75–2.20)
Age (years)		0.99 (0.94–1.04)	0.99 (0.94–1.04)	0.97 (0.91–1.02)
Female		0.93 (0.60–1.44)	0.93 (0.60–1.45)	1.03 (0.64–1.66)
Born in Sweden		0.48 (0.24–0.95)	0.47 (0.24–0.94)	0.34 (0.16–0.71)
Highest educational level: university		1.06 (0.68–1.65)	1.03 (0.66–1.63)	0.95 (0.58–1.55)
Self-reported cardiovascular disease			1.33 (0.53–3.33)	1.34 (0.50–3.62)
Self-reported hypertension			1.42 (0.81–2.46)	1.19 (0.65–2.19)
Self-reported high cholesterol			0.82 (0.38–1.77)	0.49 (0.19–1.22)
Self-reported diabetes			0.38 (0.08–1.80)	0.12 (0.02–0.93)
Poor, fairly good, good general health			0.79 (0.50–1.26)	0.62 (0.36–1.04)
Perceived cardiovascular risk higher than others				2.34 (1.28–4.31)
Referred to primary health care center				1.78 (0.98–3.23)
Referred to hospital				2.53 (1.29–4.97)
Diagnosed with hypertension and/or high cholesterol				1.36 (0.46–4.05)
Diagnosed with coronary artery stenosis				10.55 (1.97–56.37)

*Adj. OR* Adjusted Odds Ratio, *CI* Confidence Interval

Among the participants who searched for more information, 42% used the Healthcare Guide 1177 as a source of information. Participants with sufficient health literacy were more likely (adjusted OR 2.91) to use this information source compared to participants with limited health literacy (Table 4).

**Table 4:** The association of health literacy and using the national health information online portal “Healthcare Guide 1177”, OR (95% CI), *n* = 117

	Crude	Model 1	Model 2	Model 3
	OR (95% CI)	Adj. OR (95% CI)	Adj. OR (95% CI)	Adj. OR (95% CI)
Sufficient health literacy	2.03 (1.01–4.11)	2.28 (0.95–5.46)	2.09 (0.89–4.94)	2.91 (1.13–7.52)
Age (years)		1.03 (0.94–1.13)		
Female		1.37 (0.63–2.97)		
Born in Sweden		0.86 (0.29–2.59)		
Highest educational level: university		0.62 (0.28–1.37)		
Self-reported cardiovascular disease			0.43 (0.08–2.29)	
Self-reported hypertension			1.11 (0.44–2.83)	
Self-reported high cholesterol			1.51 (0.40–5.64)	
Self-reported diabetes			0.76 (0.04–14.92)	
Poor, fairly good, good general health			1.15 (0.53–2.51)	
Perceived cardiovascular risk higher than others				0.95 (0.36–2.47)
Referred to primary health care center				0.63 (0.23–1.69)
Referred to hospital				4.78 (1.53–14.90)
Diagnosed with hypertension and/or high cholesterol				1.93 (0.37–10.11)
Diagnosed with coronary artery stenosis				0.17 (0.03–0.93)

*Adj. OR* Adjusted Odds Ratio, *CI* Confidence Interval

## Discussion

In agreement with the hypotheses, this study found an association between having sufficient health literacy and I) accessing health check test results in the PAEHR and III) using the national health information online portal Healthcare Guide 1177 as a source of information, while in disagreement with the hypotheses no association was found with II) searching for information to better understand individual test results.

Our study showed that 78% of the participants had accessed their PAEHR to view of their test results. Participants with sufficient health literacy were almost twice as likely to access their PAEHR compared to participants with limited health literacy. This result corresponds with a previous research study from the US that also found that individuals with limited functional health literacy were less likely to use PAEHRs [20]. Provider endorsement has shown to increase the interest in using PAEHRs [40]. It is therefore important that health care professionals inform and educate patients and the public on how it can be used. It would be desirable for them to use methods for active learning [41], e.g., have their patients practice (with their support) navigating the website and using its various functions, including how to access their individual and/or child’s e-journal and how to think about and interpret what is written in it.

This study showed that about a third of the participants had searched for information to better understand their test results. However, no association between health literacy and seeking further information was established, which differs from previous research that found a higher level of health literacy associated with more searches for health information online [24]. This finding was surprising, since a qualitative study with the same research participants found that many expressed difficulties in understanding the test results and their implications [19]. Previous research found high risk perception and affective risk response (being worried, scared, or uncertain) to be important predictors of online health information-seeking behavior [42]. High perceived cardiovascular risk (OR 2.34, CI:1.28–4.31) and being diagnosed with coronary artery stenosis (OR 10.55 CI:1.97–56.37) were associated with searching for information to better understand the individual test results in this study, which indicate that risk perception and possible affective risk response also seem to be important factors in this context. However, the statistical models were designed for health literacy, and therefore the results should be interpreted with caution. Furthermore, most participants in this study perceived their cardiovascular risk as low [32], which may explain why fewer participants than expected searched for information.

Among the participants that searched for information to better understand their test results, individuals with sufficient health literacy were almost three times as likely to use the online national health information portal Healthcare Guide 1177. This relates to previous findings that highly educated people more often use traditional websites to search for health information, while those with a lower level of education more often use social media [43]. The fact that individuals with limited health literacy were less likely to access their test results in the PAEHR and to use the online national health information portal Healthcare Guide 1177 as a source of information is a policy-relevant finding. Since lower levels of health literacy are associated with poorer health [9, 10, 12], these individuals are more likely to need reliable health information and guidance for preventive actions. Efforts should be made to inform and direct groups with lower health literacy to these online resources. This may be accomplished by shifting focus from the individual to the organizational health literacy of the national health services, and their ability to respond to the different health literacy needs of the public [44]. Trezona et al. (2017) have developed a framework to improve the organizational health literacy. It includes to ensuring access to services and undertaking effective outreach by involving consumers in all aspects of planning, delivery, and evaluation. Furthermore, it describes strategies for ensuring that all communication is accessible and tailored to the specific health information needs and learning styles of different population groups [45]. Using sound and images can help individuals with lower levels of health literacy to understand health information [2]. It can therefore be used when educating such individuals about online resources. However, before any interventions to improve access to and use of online resources provided by the health care system are initiated, it is crucial to investigate the reasons why individuals with low health literacy do not use these resources and what health information sources are used instead.

The study uses cross-sectional data, which bring limitations regarding the causal path to health information-seeking behavior. The participants’ health literacy was measured at baseline, while the outcome variables were assessed three months later. Health literacy is also a process that can be altered through new experiences [46], which means that a participant’s health literacy could theoretically have increased by the time of the follow-up questionnaire due to factors not captured in this study. The health literacy level might therefore have been higher than what was measured at baseline, leading to an underestimation of the association. However, although the follow-up measurement was conducted three months later, the participants received their test results much sooner than that, leaving little time for changes in health literacy. The two lowest levels of health literacy were merged in the statistical analysis since there were only 15 participants in the group with lowest health literacy. It is likely that the association would be stronger for this group, but it is something that needs further research to determine. The fact that the majority of the participants were highly educated, born in Sweden, and that the age range was narrow, also puts restraints on the generalizability, although not necessarily the internal validity of the findings [47]. Since younger people search for more health-related information online [48, 49], it is not certain that the findings can be transferred to younger populations. Digital access is fundamental to enable use of any online resources but was not assessed in this study. Household internet access in Sweden is high (93.2%) [50].The design for this study, including a web-based follow-up survey distributed via e-mail, prevented individuals without internet access and computer skills from participation. Internet access constitutes a barrier that the health care system is not authorized to intervene in, which is a reason why it was not assessed in this study. This study used validated measurement to assess health literacy within a random sample of the general population and adjusted for education and other potential confounders, which is a strength of the research. The study shows that health literacy matters for health information-seeking behavior even for highly educated, native Swedes, and it contributes to knowledge about how online health services are utilized, showing that they are not reaching those who need them the most. This is an important finding, since knowledge regarding health literacy levels in the Swedish general population is scarce, and it indicates that limited health literacy needs to be considered when promoting health, and when preventing and treating health problems in Sweden.

## Conclusion

Individuals with limited health literacy do not access their personal health information nor search for health information on the online national health information portal provided by the Swedish national health care system to the same extent as individuals with sufficient health literacy. More research is needed about how the level of health literacy relates to differences in online health information-seeking behavior and how digital health information sources and e-health services can be designed to ensure that the entire population has equal access to trustworthy and quality-ensured health information.

## Supplementary Information

**Additional file 1.**

## Data Availability

The datasets generated and/or analyzed during the current study are not publicly available since they contain sensitive information, but are available from the corresponding author on reasonable request.
